# Cellular and Natural Viral Engineering in Cognition-Based Evolution

**DOI:** 10.1080/19420889.2023.2196145

**Published:** 2023-05-02

**Authors:** Miller W B Jr, Reber A S, Marshall P, Baluška F

**Affiliations:** a Banner Health Systems - Medicine, Paradise Valley, Arizona, AZ, USA; b Department of Psychology, University of British Columbia, Vancouver, BC, Canada; c Department of Engineering, Evolution 2.0, Oak Park, IL, USA; d Institute of Cellular and Molecular Botany, University of Bonn, Bonn, Germany

**Keywords:** Cognition-Based Evolution, holobiont, Natural Cellular Engineering, Natural Viral Engineering, senome, virome

## Abstract

Neo-Darwinism conceptualizes evolution as the continuous succession of predominately random genetic variations disciplined by natural selection. In that frame, the primary interaction between cells and the virome is relegated to host-parasite dynamics governed by selective influences. Cognition-Based Evolution regards biological and evolutionary development as a reciprocating cognition-based informational interactome for the protection of self-referential cells. To sustain cellular homeorhesis, cognitive cells collaborate to assess the validity of ambiguous biological information. That collective interaction involves coordinate measurement, communication, and active deployment of resources as Natural Cellular Engineering. These coordinated activities drive multicellularity, biological development, and evolutionary change. The virome participates as the vital intercessory among the cellular domains to ensure their shared permanent perpetuation. The interactions between the virome and the cellular domains represent active virocellular cross-communications for the continual exchange of resources. Modular genetic transfers between viruses and cells carry bioactive potentials. Those exchanges are deployed as nonrandom flexible tools among the domains in their continuous confrontation with environmental stresses. This alternative framework fundamentally shifts our perspective on viral-cellular interactions, strengthening established principles of viral symbiogenesis. Pathogenesis can now be properly appraised as one expression of a range of outcomes between cells and viruses within a larger conceptual framework of Natural Viral Engineering as a co-engineering participant with cells. It is proposed that Natural Viral Engineering should be viewed as a co-existent facet of Natural Cellular Engineering within Cognition-Based Evolution.

## 1. Introduction

Metagenomic sequencing studies fully establish the ubiquity of viruses [1]. For example, the marine virome is globally distributed and extraordinarily diverse [2]. A hundred liters of seawater is estimated to contain more than 5,000 viral species, and one kilogram of marine sediment has 1 million separate viral genotypes [2]. It is also acknowledged that the virome significantly shapes every aspect of biology [3]. Based on these studies, the full range of the impacts of the virome on planetary ecology and their adaptive interplay with their targets has been significantly underestimated [4]. Research confirms that the virome helps to shape the biogeochemistry of the planet by modulating microbial metabolic capabilities. For instance, nucleocytoplasmic large DNA viruses (NCLDVs) are ubiquitous across all marine environments, infect a wide range of eukaryotes, and are critical participants in biogeochemical processes in the oceans [5]. Further, the virome represents a storehouse of potential microbial genes whose fluid exchanges influence a range of metabolic and evolutionary processes [6].

Beyond this emerging understanding of the extent and influence of the virome, there is a growing appreciation that viral dynamics across the living scale are far more complex than previously supposed. Consequently, it is now accepted that viruses represent fundamental evolutionary drivers [7–12]. Notably, the prior limiting view of viruses as exclusive pathogens is being recast within this unfolding evolutionary narrative [13]. Instead, there is a growing recognition of viral symbiogenesis, which views viruses as intimate symbiotic partners that function with cells through mutualism and commensalism as well as competitive parasitism [14; 15]; [15–17]. Moreover, that active viral symbiogenesis can be productively integrated into Cognition-Based Evolution as an alternative evolutionary narrative substantially different from conventional Neo-Darwinism.

Cognition-Based Evolution (CBE) frames biology and evolutionary development as a reciprocating cognition-based informational interactome for the protection of cells from environmental stresses. Its base premise is that all living cells are cognitive [18–24]. Basal cognition is the capacity to sense the environment and discriminatingly respond to it. Prokaryotes without nervous systems demonstrate cognitive properties with associative learning and problem-solving anchoring through complex intracellular mechanisms such as the recently discovered neuron-like ribosomal r-protein networks that function like a ‘molecular brain’ [25] Pertinently, phylogenetic and structural studies indicate that these interconnecting ribosomal r-protein networks are shared across the kingdoms and have collectively co-evolved in nonrandom patterns [26]. That cognitive intelligence is required to create the nonrandom integrative information processing architecture that underlies multicellularity and its co-respondent complex panoply of cell-cell signaling strategies. Consequently, cellular intelligence represents the unifying problem-solving common denominator that operates beyond the genetic code and governs evolutionary development [20,27–36]. CBE asserts that all action in biology and evolution is driven by individual cognitive self-referential cells that receive, measure, communicate, and collectively deploy information [20,35]. In this context, multicellularity represents the coordinated assessment, communication, and deployment of information by intelligent, measuring cells to more accurately assess imprecise environmental cues. As this collective cellular action is based on the measurement and communication of information, it represents a form of Natural Cellular Engineering that serves the continuous protection of the individual participating cells [34]. Biofilms and the mixed-species tissue ecologies that comprise holobionts are their bioactive expressions. Consequently, in CBE, all multicellularity is the product of Natural Cellular Engineering and mutualizing cellular niche constructions in the continuous defense of cellular self-integrity which necessarily includes a partnering virome [37].

Within a system based on cellular cognition, evolution is coordinate cellular problem-solving. Genes are not direct evolutionary drivers but are flexible tools of intelligent cells in their confrontation with environmental stresses. Consequently, evolution is not determined by random genetic variants. For instance, recent studies in the plant Arabidopsis thaliana confirm that there is mutational bias that reduces mutations substantially within essential genes, coaligning with a demonstrable epigenome-associated bias [38]. Consequently, mutation can no longer be considered a directionless force in evolution. Instead, variations arise from epigenetic impacts that drive differential patterns of Natural Cellular Engineering through largely nonrandom genome editing, producing altered phenotypes [39]. Accordingly, the operative propulsive drive in evolution is collective cellular action to assimilate environmental challenges. Consequently, natural selection can now be appropriately appraised as a post-facto filter of preceding cell-based variations [34]. Natural selection assures that those cellular-based measurements of environmental cues deployed through engineering are in synchrony with the external environment, applying at all scales, whether biofilms, the localized tissue ecologies within holobionts, or holobionts as a collective entity.

When cellular cognition predominates, the maintenance of individual cellular self-identity is paramount. That maintenance of self represents the continuous internalization of the external environment dependent on the coordinated measurement of environmental cues through multicellular cooperation [34,40]. Consequently, evolution can be recast as a reciprocating cognition-based informational interactome in perpetual cellular confrontation with environmental stresses [20].

A further extension of CBE is now proposed. The virome should be considered a full co-participant in Natural Cellular Engineering and natural genetic engineering (genomic editing) through a corresponding process of Natural Viral Engineering (NVE). Evolutionary development is thereby placed within a cohesive and encompassing framework based on the energizing and reciprocating processes of natural engineering and mutualistic niche constructions as ongoing perpetual symbioses among Prokaryota, Archaea, Eukaryota, and the virome [35].

As a necessary codicil to this argument, exchanges between cells and viruses require thorough reconsideration beyond the traditional host-parasite model. Further, the assumption that viral quasi-species generation results from random genetic variations is called into question. Instead, most virocellular interactions are transfers of nonrandom recombinant genetic modules that confer bioactive cellular expression as part of a universal biological process of Natural Cellular Engineering [39]. Reciprocally, cellular genetic material represents its own source of modular substitutions for recipient viruses as part of continuous virocellular exchanges. Therefore, it can now be proposed that multicellularity represents viral-cellular co-engineering as a continuous, synchronous interplay among the cellular domains and the virome. The drive of this consistent reciprocity is the continuous endogenization of the planetary environment for the perpetuation of each of the four domains.

## 2. Current concepts of the virome

The Four Domains can be divided into two major categories: ‘capsid-encoding organisms’ (viruses) and ‘ribosome-encoding organisms’ (cells) [41]. Raoult and Forterre [42] have proposed terming capsidless viral elements such as mobile elements, plasmids, or transposons as ‘orphan replicons’. Just as the cell membrane circumscribes the cell and is a major aspect of cellular capacities, capsids are sets of proteins that encapsulate viral nucleic acid and are an integral part of the viral apparatus. Within these defining terms, the basic organizational structure of the virome can be interpreted as having a conceptual overlap with cells. The genetic materials of cells (central genome, mitochondria, the variety of RNAs, and circular DNAs) and viruses (encapsulated viral genetic material) relate to their respective boundary structures. In the case of cells, there is recognition that their boundaries are an integral aspect of their problem-solving capacities [43]. Viruses encode their capsids which serve a correlative role in viral dynamics that the cell membrane evinces in cellular dynamics with its own flexible particulars. Studies indicate that capsid proteins have originated from diverse cellular host ancestors [44,45]. So-called giant viruses generate huge capsid shells harboring up to 1.2 Mbp-large genomes [46–49]. In addition, enveloped viruses steal membranes from their target cells [50,51].

Forterre [52] notes acutely that viruses have been typically defined by their virions, arguing that this classification defaults to the timeline of discoveries rather than viral properties. Bândea [53] suggested differently, asserting that a virus is a dual-stage organism with two distinctive phenotypic phases: 1) a vegetative phase, dispersed within cells that begins the synthesis of viral proteins and exhibits viral metabolism, growth, and reproduction, and 2) the phase encompassing the reproductive reassembly of nucleic acids, viral particle sub-components including receptor proteins, and some additional cellular molecules to complete a “protective layer”, comprising an entire virion. In this model, virions are viral spores suited for continuation outside of the cell, and the virus proper is the infected cell. Thus, viruses are complex living entities that infect cells and transform them, expressing their actual viral character [54]. In this view, the virus makes virions as one ‘life-cycle’ stage, and the novel organism that results from transfection is a ‘virocell’ ([41,55].

Nasir et al. [56] contrast the two stages of the viral reproductive cycle: an intracellular stage in which the virus reprograms a cell to reproduce virions and an extracellular stage in which the virion exits the cell and enters the external environment. The latter is most frequently identified as the ‘virus’ since it is technically feasible to count these after purification. They argue it is the intracellular phase that produces a virion factory that “better represents the ‘virus self’” and virions are simply means to “disseminate genetic information much like human gametes and plant seeds” [56].

It is increasingly recognized that the properties of cells and their viruses as ‘virocells’ can overlap. Viruses can compartmentalize some of their functions, including genome replication and particle assembly [57]. Those compartments may contain cellular resources such as organelle membranes, or instead, may be comprised of viral proteins. Such compartments have been termed inclusion bodies, viroplasms, and viral factories, subject to many varieties. This kind of division of labor by viruses within their cellular context, utilizing disparate resources of varying intracellular origin organizing and assembling into a functioning product, should be considered its own form of natural engineering in which capsid envelope proteins participate.

Krupovic and Koonin [45] indicate that many capsid proteins, and perhaps all, have a cellular origin derived from the ancestral proteins of cellular organisms. However, these same viral proteins can self-assemble within cells into virus-like particles (VLPs) as supramolecular complexes that resemble complete viruses [58]. These virus-like particles are not infectious since they contain no viral genome, but they can induce innate and adaptive immune responses comparable to those of complete viruses. Formed by nucleocapsid proteins, the final shape of VLPs tends to resemble the symmetry of the original parental virus [58]. However, they exhibit more variety as their final configuration depends on the resources available during their *in situ* cellular assembly. Remarkably, since a viral genome is absent, VLPs will package nucleic acids (RNAs) within target cells as part of a complex pattern of expression, which leads to further induction of humoral immunity. Given the variability of VLP assembly based on available cellular resources including cellular nuclei acids, it can be construed that viruses can interact with cells in the same manner that cells use their own resources as tactical tools. In turn, human bioengineers are skillfully assembling and deploying VLPs for vaccines and leveraging their therapeutic delivery as our tactical tools [59].

Pertinently, human bioengineers have determined a practical function for their purposes, but it is unclear how VLP assembly benefits viruses. If nature is parsimonious, VLPs must be serving a role insofar as it is a conserved complex process exhibited by an extensive range of viruses, surviving eons of selection. One possible justification is that VLPs serve as a vehicle that fits into the complex co-engineering patterns between viruses and cells whose exact role is yet to be determined. Indeed, its precise role might be indirect. For example, extracellular vesicles (EVs) are an essential component of cell-cell communication. Viruses can utilize EVs to modulate the immune capacities of uninfected cells and evade cellular immune systems, thereby establishing or maintaining chronic infections or deterring the actions of competitor viruses [60].

Despite a growing literature about viruses in their specific actions within cells, their interrelations across a broad ecological context remain little examined [61]. Recent studies have uncovered that phages can reprogram bacterial cell metabolism, contributing different ecosystem-wide potentials with extensive planetary consequences, including affecting target cell metabolisms and translation machinery. Global-scale viral metagenomic studies indicate that viruses can encode ribosomal proteins in cells, contributing to mutualizing adaptations [62]. Viral genomes, including those of giant viruses, have contributed actin-related genes to eukaryotic hosts, assisting in cytoskeletal development [63].

Ocean viruses are abundant and infect a significant percentage of surface microbes, with the potential for repressed host transcription and the reprogramming of carbon and energy metabolism [61]. Advances in metagenomic sequencing technologies indicate a vast forest-wide virome of enormous depth and complexity. A permanent multilevel interplay between viruses, trees, shrubs, and other microbes governs forest health in which there is constant feedback and reciprocation among all forest participants [64]. Thus, the concept of the virome should be expanded beyond that of obligatory parasites whose actions are a consistent set of defaults enacted through a mechanical co-option of cellular machinery. Instead, the virome plays a commensurate role with cells as active biological players capable of complex, directed, and effective intracellular and extracellular actions [7,10–12,16].

## 3. Origin of viral genes

The origin of viral genes remains controversial. Some assert that most new viral genes arise *de novo* in viral genomes, occurring during the intracellular viral reproductive stage in much the same manner that it has been long assumed that cellular genes originate [54]. Moreira and López-García [65] contended differently: “All in all, viruses are gene robbers, not gene inventors and massive gene suppliers” [65]. It is believed that the dominant genetic flow is cells to viruses, so most of the viral genes that can become further involved in cellular energy and carbon metabolism, transcription, and translation were acquired by viruses through horizontal gene transfer (HGT) [65]. Further, on multiple occasions, it has also been found that viral capsid proteins have evolved from ancestral cellular proteins [45].

More recently, Nasir et al. [56] noted that viral genomes are predominated by virus-specific genes that do not have cellular homologs. Consequently, it has been proposed that viral genes originate as genetic segments derived from short open reading frames (protogenes), or in the case of RNA viruses, from non-coding strands of ancient genes as templates that yield de novo genes [41]. For example, in the bacteriophages in the family *Microviridae*, novel genes have been traced to preexisting sequences as over-printing areas of genetic overlap, accounting for a gradual increase in complexity of the phage genome [66]. Therefore, fresh viral protein-coding genes can originate either de novo from overprinting or by modifying existing viral genes, explaining why many viral genes do not have cellular homologies [67]. Yet, as Forterre [41] emphasizes, this viral creation of new genes represents a vital cellular resource.

Throughout evolution, viral segments have commonly integrated into cellular chromosomes. Although it has been assumed that the composition of these segments is random, it is noteworthy that many viral segments are modular, appear repetitively, are subject to transfer among the cellular domains, are often conserved [68], and can be a source of novelty and evolutionary creativity [39]. Indeed, Witzany [69] argues that the longstanding narrative of evolution through replication error should be replaced with a different perspective concentrating on genetic content editing involving modular transfers and recombinations through the action of nonrandom genetic content editors. Viruses and transposons share common properties. The horizontal transfer of the latter and their high levels of translation and transcription provide opportunities for transforming cellular regulatory gene networks, representing feed stock for the origin of new viral genes or viruses themselves [70]. What is no longer controversial is that genetic transfers and the exaptation of viral proteins have contributed to major evolutionary transitions [71,72]. Thus, the contentious issue of the origin of viral genes reflects the complexities invoked by the fluidity of genetic transfer among viruses and cells across evolutionary space-time.

## 4. Genes and vesicles interchange across the domains

It is no longer disputed that viral genetic transfers are essential elements of our planet’s evolutionary narrative. These exchanges serve as sources of exaptations for various cellular functions, as components of antiviral defenses, and stimulate nutrient storage and further gene transfers [72]. Nor is it disputed that HGTs are common in the unicellular realm. Delavat et al. [73] cite substantial evidence that “many if not most prokaryotic genes have at some point been horizontally exchanged between species” [73]. Many of these can be subject to pervasive inter-domain transfer. The development of many crucial metabolic pathways in eukaryotes has been dependent on HGT, such as critical nitrase reductase for nitrate assimilation. This metabolic pathway has been supported by three bacterial gene transfers that contributed to the origin of the pathway and seven further transfers among eukaryotes, displaying a reticular evolutionary pattern [74]. Similarly, HGT is acknowledged as an essential element in archaeal adaptation and diversity by both a flow of Integrative Conjugative Elements (ICEs, also known as integrative transposons) that contributed to archaeal cell wall anatomy and metabolism and viral genetic transfers [75,76].

Until recently, the dynamics of this interchange was believed to be haphazard and random. This prior narrative has now been supplanted. Prochlorococcus is an ocean-dwelling cyanobacterium that lives by photosynthesis. Surprisingly, their cyanophages carry photosynthetic genes [77]. Phylogenetic studies demonstrate multiple back-and-forth exchanges of these genes between the cyanobacteria and their phages. These exchanges have been essential for their niche stratification into two dominant, separated oceanic niches [78]. A high-intensity-light ecotype that evolved 150 million years ago exists near the surface. A low light-intensity ecotype thrives at lower sea levels and evolved more recently. Both adaptations required novel genes that were repeatedly distributed through horizontal transfers. Shih and Goldenfeld [78] conclude that this phenomenon is an example of multi-scale ecologically-based feedback, driving evolutionary complexity and ecological stability through collective, mutualizing, and rapid evolutionary shifts to meet environmental change. This conjoining process is characterized by collective sensing and decision-making, permitting co-evolutionary benefits for both the bacteria and the phages. The bacteria represent a “slowly evolving, stable repository of beneficial genes for phages by filtering out inferior genes under selection” and the phages offer “a rapidly evolving reservoir of genes for the host bacteria” [78]p. 30), Crucially, these reciprocations represent a flow of information that goes beyond the predator-prey dynamics which has long been supposed as the operative evolutionary mechanism.

In recent years, there is a growing recognition that HGT is a significant and frequent phenomenon in Eukaryota [79]. Although HGT is generally considered more common from cells to viruses with the exception of retroviruses, there is widespread transfer of capsid protein and RNA-dependent RNA polymerase genes from double-stranded RNA viruses to eukaryotic nuclear genomes [80]. This interplay is typically considered in the context of virus-host co-evolution, involving the transfer of a range of endogenous genetic modular segments, viral genes, or even entire viral genomes [81]. Most originated from retroviruses, but multiple instances of non-retroviral integrated RNA virus HGT have been documented and may be more common than previously acknowledged [80]. None of the cellular domains are exempt. It has been known since the 1980s that specific classes of viruses preferentially infect archaea [82]. To date, the archaeal virosphere has been sparsely explored, but it is clear that archaeal phages are globally distributed [83,84]. Moreover, in Eukaryota, endogenous viral elements have played an essential role in the formation of the viral protein envelope of the placenta in lizards [85]. The syncytin genes necessary for mammalian placenta development originated in viruses, and over 50% of the human genome consists of endogenous retroviral elements [81].

Recent research confirms that prokaryotic genes are subject to frequent HGT in eukaryotes such as algae, serving important ongoing roles in eukaryotic adaptation including polysaccharide biosynthesis and heavy metal detoxification [86]. Further, a small but significant proportion of human DNA sequences originate from bacteria and other microorganisms [87]. At the same time, retroviruses serve as intermediaries between prokaryotes and eukaryotic cells among holobionts. Further yet, commensals and immune cells communicate through endogenous retroviruses [88].

Viruses receive insertions of mobile DNA from many sources. For example, the genome of the megavirus *Pandoravirus salinus* has been invaded, colonized, and shaped by MITEs (Miniature Inverted-repeat Transposable Elements) which are short-length non-autonomous DNA transposons whose transposition enzymes are encoded by other autonomous DNA transposons [89]. These invasions can result in multiple copy insertions assumed to have played a significant role in pandoravirus genome evolution [90]. Reciprocally, however, some of these transfers can go in the opposite direction. For example, MITE transfers have been shown to emanate from amoeba to their viral parasites and from the wasp genome to pandoraviruses. Unquestionably, the lines blur. Many viruses can act in the same capacity as mobile DNA elements through either temperate bacteriophages, retroviruses, and other tumor viruses, and further, they are capable of encoding DNA restructuring [91,92].

The viruses of the *Baculoviridae* family infect a moth. There are a large number of diverse moth DNA sequences integrated into that viral family via transposable elements [93]. It is presumed that these transfers came from HGT from the baculoviruses of other insect species. Interestingly, none of these genetic signatures persist beyond ten successive infectious cycles. Renner and Szpara [94] note that at least 20% of the core genes shared among herpes virus subfamilies have cellular origins. Others have originated in other viral species, attesting to the fluid dynamics of gene transfer among the domains. Indeed, the chromosomal germline integration of human herpesviruses (ciHHV), typically HHV6A, is detected in about 1% of the human population [94].

Viruses play a crucial role in the evolution of novel protein functions, proposed to represent modifications of preexisting cellular ‘orphan genes’ [65]. Yet, systematic analysis shows that fewer than 3% of prokaryotic orphan genes have viral homologs, concluding that viruses are not prolific donors of cellular genes but, instead, serve as a reservoir of transferred cellular genes that can become available from different host targets. Metagenomic research on nucleocytoplasmic large DNA viruses (NCLDVs) suggest even greater levels of complexity. The large gene repertoires of these giant eukaryote viruses encode for many metabolic genes and pathways, including fermentation, nitrogen metabolism, sphingolipid biosynthesis, glycolysis, and gluconeogenesis [95]. Some of these genes are believed to have been acquired by HGT from cells, but others seem to have distinct evolutionary lineages. What does seem clear is that NCLVDs are active metabolic co-participants within a cellular context, mediating metabolic reprogramming in keeping with the virocell concept.

It has been long recognized that virus genomes are mutable through genetic transfers. Tissue culture experiments have documented that viral genome recombinations are frequent, contributing to viral adaptation and virulence [49]. These recombination events are thought to be confined to inter-cellular replication. However, recombination between viral lineages occurs through cross-species transmission at a frequent rate, accounting for substantial viral diversity. Hence, virus-to-virus genetic transfers are a significant factor in the phylogeny of viral lineages and also has specific consequences for human pathogenesis [96].

More recently, other inter-domain genetic transfer mechanisms have been identified. Trans-kingdom small RNA (sRNA) transfers are abundant, triggering consequential interactions between parasites and targets [97,98]. Further, various non-coding RNAs, including small interfering RNAs and microRNAs, have been identified as participating in cross-species and inter-kingdom communication, particularly among corals, plants, and mammals [99]. These contribute to environmental sensing, gene expression and genetic regulation, host defenses against viruses and potential parasites, and a wide variety of other biological functions [100]. Recent research demonstrates that non-coding microRNAs participate in the inter-domain cross-talk between the participants in the gut microbiome of mammals, including humans [101]. There is even evidence that diet-derived microRNAs can contribute to genetic transfers and the modulation of gene expression in mammals [102].

As a further means of inter-domain genetic communication and cross-talk, microbial extracellular vesicles are known to package small, non-coding RNAs that can enter the cytoplasm of target cells and influence gene expression [103]. Thus, these extracellular vesicles are significant factors in microbial communication and trans-kingdom genetic exchanges.

Based on observations of megavirus activity, a picture of a complex genetic interplay emerges that suggests an accordion-like model of viral evolution. Viral genomes have undergone intermittent and successive genetic gain and loss [104]. As a result, mobile genetic elements and giant viruses form their own type of mutualistic symbiosis, similar to the relationship between the virome and their respective transposon transcripts [105]. Therefore, the evolutionary histories of viruses and transposable elements are tightly linked [106]. DNA transposons are known to contribute to viral genetic novelty. Many eukaryotic viruses, plasmids, and transposons connect through a shared gene pool [45]. In this manner, eukaryotic genomes have been likened to ecosystems inhabited by a diverse community of transposable elements capable of exchanges within cells in nonrandom patterns [107]. Similarly, trans-kingdom exchanges in which the virome is a crucial intermediary can be considered part of a continuum of a planetary-wide genetic ecosystem in which the virosphere participates within a deeply entangled network of gene sharing [108]. Ameobas remarkably illustrate the fluidity of these types of trans-kingdom exchanges. Many diverse bacteria and viruses multiply naturally in amoeba, including the giant NCLDV Marseillevirus whose genome includes a mixture of NCLDV core genes, genes from eukaryotic hosts, and others from varied eukaryotic bacterial and viral symbionts [109]. Consequently, it has been proposed that amoebae should be considered “melting pots” of trans-kingdom evolutionary exchanges [109,110].

Therefore, the virome is a vital intercessory of communication and biological capacities across the four domains as a salient adaptive pathway among holobionts [35]. Large-scale gene-species phylogenetic tree reconstructions of HGT-derived genes of microbiota isolated from six major human body sites have been conducted by the NIH Human Microbiome Project [111]. That study confirmed that HGT activity increases significantly in the genomes of inhabitants of the human microbiome. More than half of the genomes of human-associated microbiota have been transferred by HGT across multiple body sites. Furthermore, as one feature of this ample process, it has been shown that competent bacteria can take up extracellular DNA and utilize it [112]. Various mechanisms are utilized, including conjugation, bacteriophage-mediated transduction, or via gene transfer agents such as phage-derived encapsulated DNA sequences or outer membrane vesicles that can encapsulate DNA [112]. Through multiple means, viruses exchange genes with each of the cellular domains through established non-lytic interactions with cells and by endogenizing into the genomes of bacterial endosymbionts that reside in eukaryotic cells [113]. Such interactions create opportunities for extensive genetic exchanges between viruses and organisms of non-host organisms. Thus, genetic affiliations between bacterioviruses and Eukaryota, and eukaryoviruses and Prokaryota are no longer surprising. Nor is it unexpected that there is a large cohort of protein folds universally shared by viral and cellular proteomes or that virus-specific protein folds are not detectable in cellular proteomes [113].

## 5. The role of viral modularity

Botstein [114], proposed a modular theory of virus evolution, conceptualizing viruses as belonging to interbreeding families whose members share a common thread of inherently modular genomic organization. Interchangeable elements crucial to specific biological functions can be fluidly traded among organisms, particularly temperate phages [114]. Research in the last few decades confirms that modularity is characteristic of biological systems and confers performance advantages [115]. Efficient compartmentalization within genetic regulatory networks permits the adjustment of an isolated genetic component without engendering system-wide disturbances [116]. Genetic and protein structure and biological networks have examples of modular partitioning that contribute to metabolic, genetic, and protein-protein interaction networks [117]. The central role of modularity within the gene regulatory networks that underlie developmental processes and body plan patterning is also acknowledged [118]. As a result, modularity is regarded as a central concept in evolution that enhances our understanding of adaptive landscapes and serves as a potential bridge between micro and macroevolution [119].

The role of modularity extends beyond any molecular or genetic mechanisms. The same principle applies to microbial life-cycle progression, specifically involving patterns of motility and sporulation that act as discrete units [120]. This same universal biological principle of efficient modularity also applies to the virome [68]. Koonin and Dolja [68] assert that homologous capsid proteins are represented in a great proportion of icosahedral viruses that infect each of the three cellular domains and combine with widespread hallmark genes specific to the viral replication apparatus. Thus, viral evolution has an “inherently modular character” [68]. Koonin et al. [106] indicate that the eukaryotic virome is distinct from that of prokaryota. The eukaryotic virome is dominated by RNA and retroelements as the product of extensive modular gene exchange, some of which can be traced to prokaryotic ancestry. Recent reconstructions of the evolution of the global RNA virome that dominates in eukaryotes indicate that its breadth has been significantly underestimated. There have been extensive transfers of viruses among distantly related plant and animal hosts through extensive modular genetic exchanges and horizontal transfers [121].

Human adenovirus D (HadV-D) is a significant pathogen with high levels of diversity [122]. Recent studies of HadV-D attribute this diversity to high rates of intertypic recombination between distinct types. Specific genomic regions are conserved in this process as “universally conserved segments” (UCSs) that facilitate homologous recombinations. Segmental exchanges target USC-flanking genomic regions and transfer as recombination modules that result in reshuffling and chimeric genomes similar to the mechanisms within major histocompatibility complexes in vertebrates. This modularity surely factors into the preservation of UCSs given their persistence. Further, cetacean papillomaviruses have a core modular genome composed of four highly conserved genes that can undergo insertions or deletions of genetic elements representing forms of interspecies diversification [123].

The factors that influence modular transfer are still little understood. However, it is known that the negative sense ssRNA genomes of influenza viruses are segmented into eight parts within their virion and are much more likely to undergo segmental exchange in the context of cellular co-infection. Cellular co-infection by two or more different influenza viruses produces segmental genetic exchanges in a process that has been labeled antigenic shift [87]. It is believed that these types of antigenic shifts of virus-to-virus recombinant reassortment of entire gene segments as modular components were consequential to the virulence of the 2009 H1N1 ‘swine flu’.

Modularity represented at the genetic level through segmental transfers must necessarily impact the proteome to exert biological expression. Research indicates that modular protein interaction networks are significant determinants of the mode and tempo of evolution through patterns of constraint and plasticity in which proteomic shifts have their own modular character [124].

Given the meaningful role of modularity in biological networks, a further derivative arises. Blind mutations cannot be the progenitors of modular networks or any propensity for modular exchanges. Once modularity is appraised as a valid mechanism in biological networks, there is no reason to suppose that these *in toto* segmental exchanges would be random. And further, given that these are en bloc genetic transfers, it follows that there is no requirement that the resulting biological shifts yield merely gradual differential physical expressions [125].

## 6. The role of the viral capsid

With the division of biology into two principle features comprised of viral capsid-encoding organisms and cellular ribosome-encoding ones, their continuous genetic interplay becomes the major source of biological evolution [8]. Although the virion is typically framed as the defining element of viral architecture, Koonin and Dolja [68] assert that viral self-identity relies on the composition of the capsid, which denominates its actual deep ancient lineages. This reinforces dividing the virome into capsidless genetic elements such as plasmids and a wide variety of transposons and capsid-encoding viruses. All of these share hallmark genes, and each can serve to create one another [68].

Viral nucleocapsid proteins encase viral genomes and act as a templating participant in viral replication and transcription [126]. They serve essential roles in viral assembly by interacting with target cell proteins and disrupting cellular defenses, such as interferon stimulation. Recent research in Middle East respiratory syndrome coronavirus (MERS-CoV) confirms that nucleocapsid proteins participate in the active upregulation of multiple viral genes in infected cells at the transcriptional and translational levels, dependent on specific nucleocapsid domains and motifs [127].

In addition to their nuclei acids, viruses are carriers of various capsid proteins, often linked to lipids or sugars [128]. Each of these is a participant in a highly complex communication process with the target cell supporting the release of the viral genome. The uncoating of that genome is an interactive process in which the target cell provides many of the molecules and cues for uncoating and directs the virus to the site of replication which may be in the cytosol, cytoplasmic membranes, or nucleus [128]. Thus, viral uncoating and infectivity are the result of combined action among viral and cellular receptors, enzymes, and cell-based chemicals that alter capsid structure and direct replication.

## 7. Virocellular interactions as co-engineering and mutualizing niche constructions

The impulse toward multicellularity is based on the conditional ambiguity of biological information [35,37,40,129,130]. Multicellularity permits the collective supracellular/cellular measurements of environmental cues as a ‘wisdom of crowds’ assessment by multiple different observer/participants through shared cell-cell communications [34,35,40]. Therefore, any genetic transfers are information exchanges, and in that context, genes must be considered tools of cells [20,34,35,40].

The spread of antibiotic resistance across a biofilm through directed horizontal gene transfer of antibiotic-resistant genes is an example [131]. In multiple bacterial species such as *Bacillus subtilis*, HGT by conjugative plasmid ICEs is crucial for spreading antibiotic resistance [132]. Multicellular biofilm formation encourages this type of conjugative transfer through the mutualized production of extracellular matrix substances essential for that transfer. *Bacillus subtilis* can express two types of extracellular matrix. One is an effective extracellular matrix for cell surface adhesion, and the other is optimized for colony mobility [133]. Which matrix is deployed depends on coordinated sensing of environmental surfaces as smooth or irregular. Matrix production depends on sub-specializations among the participants to enable productive co-engineering [134]. These sub-specializations result from consensual partnerships requiring that some cells voluntarily lose some of their cellular functions to streamline the production of matrix. Consequently, those cells are dependent on a replacement of that deficiency through traded resources. Through this negotiated compact, some cells are placing their survival in the hands of other biofilm constituents to reach mutual goals.

Horizontal transfers among cells and viruses proceed by a variety of mechanisms. Integrative and conjugative elements (ICEs) are comprised of various widespread mobile DNA elements as segments of prokaryotic chromosomes that can transfer independently of phages or plasmids [73]. ICEs exist in either an integrative state, remaining within a cellular chromosome and subject to vertical transmission, or a conjugative state, in which the DNA is edited from a chromosome and becomes available to conjugate in another cell as horizontal transmission [73].

**Figure 1.**
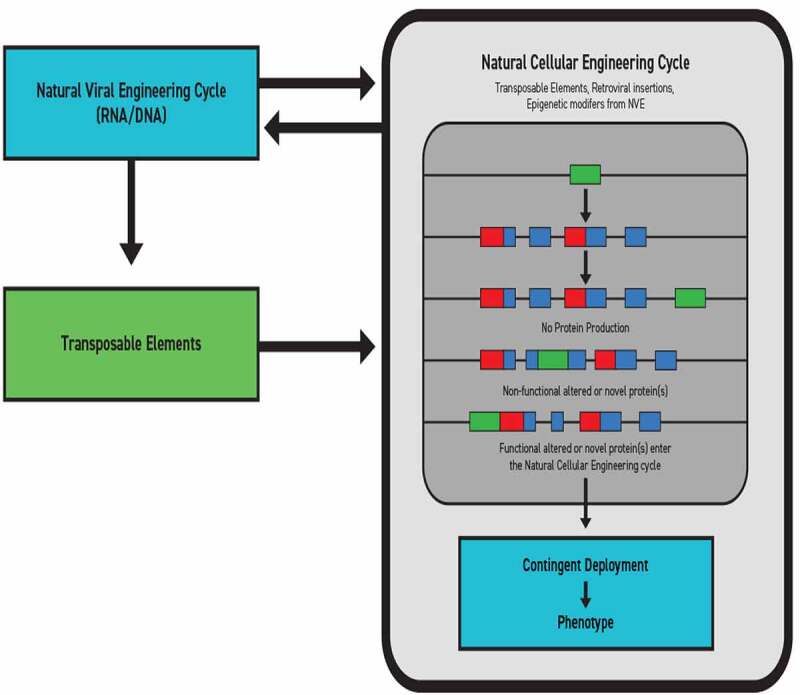
**Viral-cellular engineering cycle.**Co-engineering in Cognition-Based Evolution is based on a co-respondent relationship between NVE and elements of genetic exchange, such as transposable elements, that reciprocate with a Natural Cellular Engineering cycle. Sequence insertions or deletions yield variable proteomic outputs, either unchanged protein production, a new nonfunctional protein, or a functional or novel protein capable cells can deploy as phenotypic variation.

In the past, each event was regarded as a separate phenomenon that achieved coordination absent any annealing driver other than natural selection. However, natural selection is merely an environmental filter of preceding actions and provides no intrinsic propulsive force [23,34,35,40]. Given the cascade of reciprocations and collaborations required to effect shared genetic transfers in the highly complex environment of biofilms, there is a compelling need to place all of these manifold exchanges into one concordant framework. It is argued that such complex coordinate processes imply purposive action. Consequently, genetic transfer across established networks in response to collectively assessed stresses should be regarded as an example of natural genetic engineering as a feature of Natural Cellular Engineering and mutualized niche constructions. Its nonrandom direction is the consistent drive toward continuous organismal-environmental complementarity [34,39]. In the case of the transfer of antibiotic-resistance genes in biofilms, the genetic exchange is of viral origin, and the phage is an active participant. Since this is a reciprocating process between cells and viruses, it defaults that there is a mirroring form of concomitant Natural Viral Engineering acting pari-passu with Natural Cellular Engineering (Figure 1).

A claim that viruses engage in any form of natural engineering would entail providing evidence that viruses can substantially mimic cells in having some capacity for contingent decision-making. This claim can be supported by the accumulating evidence that viruses, like cells, have a palette of limited social abilities. Over the last few years, abundant information has accumulated indicating that viruses can act in their own behalf by communicating and cooperating both outside and inside cells [135–137]. Consequently, these exhibited faculties should be considered as a form of limited contingent decision-making and problem-solving.

When a temperate phage adsorbs into a target cell, it can either initiate a lytic cycle or become a dormant prophage. As Ofir and Sorek [138] indicate, the phage must make a lysis-lysogeny ‘decision’ coordinating through an intricate network of transcriptional repressors or activators and additional processes involving RNA degradation and proteolysis. Such a distinction between two such differing pathways must be determined by viral reception and interpretation of information that assesses both the metabolic state of the cell and the presence of co-infecting phages. It follows that this complex activity requires measurements of status. The concentration level of a six amino acid long peptide contributes to this lysis-lysogeny decision pathway as part of a collective viral assessment and communication system [139]. The peptide forms part of an ‘arbitrium’ communication system of three phage genes: *aimP* used for peptide production, *aimR* for peptide reception, and *aimX*, which serves as a negative regulator of lysogeny. Further, this reciprocating system produces information that is communicated among successive populations of intracellular phages [139]. It has been previously maintained that phages can receive information, distinguish between ‘self’ and ‘non-self’, cooperate or compete, communicate with each other, and coordinate contingent actions [137]. However, such actions can only occur through information measurement. It follows that since viruses can measure, communicate, and selectively deploy information, they are capable of participating in co-engineering with cells.

## 8. Viral impacts on cellular senomes: from sensory viral-host navigations to sociovirology

The ‘senome’ is defined as the summation of all the sensory inputs of cells that contribute to information reception and assessment [43]. For cells, the senome is the central gateway for the reception and cognitive assessment of information that can be collected and channeled from the external environment, forming a critical aspect of the cellular information management system [34,37,40,43,140]. Therefore, the senome is the informational nexus for cells, connecting its entire sensory apparatus to its plasma membrane. That sensory reception and memory are encoded within a plethora of bioactive molecules and genetic tools within the separate cellular compartments with a surprising depth of complexity. Studies in cellular protein synthesis indicate that ribosomal protein networks participate in the sensing and transfer of information, contributing to cellular decision-making by functioning in a role equivalent to nervous systems [141]. Similar to neurons, ribosomal “sensory-proteins” form “molecular synapes” that interconnect information processing via simple “molecular brains” that interface and coordinates from cellular sensory cues into coordinated protein expression [142].

Viruses lack an excitable plasma membrane but assemble a capsid protein shell. Alive or not, the fact that viruses can engage in a broad suite of complex contingent actions suggests that viruses also have a quasi-senome of their own type, representing their means of engagement with the environment. Thus, our contention is a viral quasi-senome permits viruses to experience their environment as its interface, enabling reciprocating interactions between the viral capsid, its genetic memory, and its virion with its environmental surroundings.

Indeed, the senomic capacity of the nucleocapsid has been manifestly demonstrated. Virosomes are reconstituted viral envelopes that have been bioengineered by removing nucleocapsids from enveloped viruses [143]. These virosomes can be used to actively transport drugs or vaccinal antigens, as they retain their ability to fuse and bind with target cell membranes. They also selectively deliver the virosomal package into the target cell cytosol. This independently retained faculty argues for applying the senome model to the viral realm, encompassing the complex senomic capacity of the arbitrium system and its memory component. Therefore, viruses link to information space through their senomic system, which provides a requisite coordinating link to the environment, activated through a proscribed sense awareness. Crucially then, viruses must be deemed active sensing agents of any existing symbiosis with cells rather than passive passengers or mere default pathogens.

Further, the senomes of cells and quasi-senomes of viruses necessarily link, thereby representing their engineering interface. For instance, prophages can serve as bacterial regulatory switches. A temperate phage can integrate into a bacterial gene and disrupt its function, yet, based on the bacterial assessment of information, it can be excised from that gene, thereby restoring its function [138]. Moreover, prophages are abundant residents of bacterial genomes, representing a crucial component of the cellular viral defense system that blocks destructive infection by either temperate or lytic phages [144].

Furthermore, insofar as cells are viral targets and appropriate ones are readily found, it can be imputed that the cellular senome and viral quasi-senome could have an attractional effect, potentially guided by extracellular episenomic and bioactive/bioelectric fields. Further, on cell entry, viral-cellular senomic overlaps stimulate co-engineering, enabling viruses to act as part of a creative, living virocellular system.

**Figure 2.**
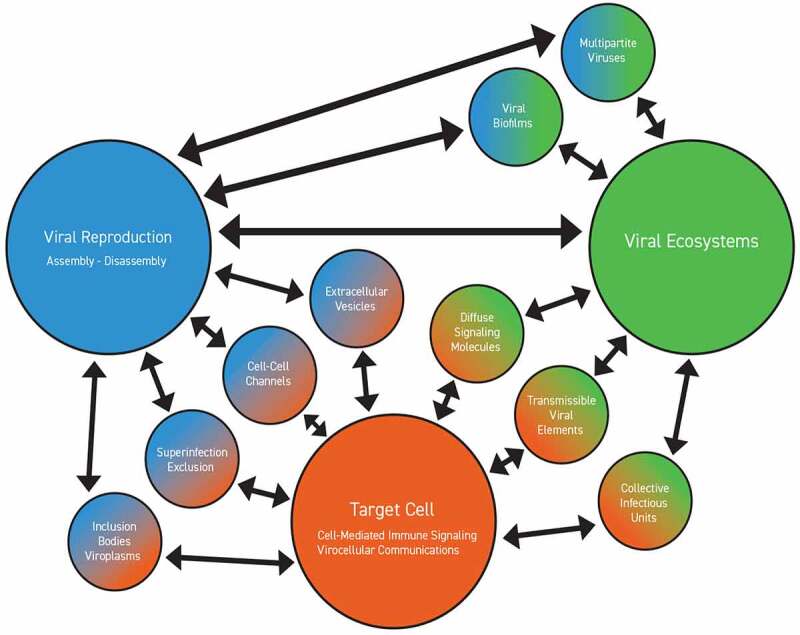
**Sociovirology, viral ecosystems, and virocellular communications.**Viruses exhibit extensive social attributes, enabling coordinate virus-virus and virocellular communications through multiple signaling pathways. Collectively, these enable the lytic-lysogenic decision matrices of viruses, enacted through the social principles of collective action, co-dependence, and competition that underpin viral ecosystems.

Just as with cells, viruses exhibit a variety of communicative and ‘social’ behaviors. Some viruses rely on ‘helper’ viruses to complete a lytic cycle, yet others suppress those activities in the presence of viral co-infection [136]. At times, viruses cooperate to permit co-transmission as a combined infectious unit. Viral genomes can differentially promote other viral genomes or selectively block them. Viruses and sub-viral particles can produce target-cell immune changes that promote viral transmission or induce the production of molecules essential for that transmission to benefit other viruses [145]. Further, viruses can produce viral proteins that communicate cellular infectious status to other viruses or induce target cells to produce signals for distant cells that expose receptors favoring viral entry for preferred partners [136]. These overlapping capacities all require varying levels of communication, cooperation, and competition, termed ‘sociovirology’ (Figure 2).

Complex cooperative virus-to-virus interactions can produce cohesive viral units that can be considered viral biofilms. For example, T-cell leukemia type 1 viruses will cocoon themselves in a carbohydrate-enhanced adhesive extracellular matrix which protects the viral population during transfer between cells [146]. This communally engineered architecture resembles bacterial biofilms, representing cohering, cooperative viral communities. In addition, many viruses have been identified that form collective infectious units that permit the joint dispersal of viral genetic materials [145]. This combined social assessment of cellular status through virus-virus interactions extends to the evasion of cellular interferon-based innate immunity. Interferon-stimulating viruses will assess the status of neighboring viruses to determine whether interferon pathways should be blocked [147]. This decision matrix can yield a loss of viral progeny production which has been termed ‘altruistic’ in an evolutionary sense [147,148].

The suite of viral social characteristics is remarkably similar to that of cells. Viruses chatter extensively among themselves to support contingent mutualized outcomes [149]. Bacteriophages are demonstrated to collaborate, compete, co-link to share resources, use host resources prudently, and sometimes cheat [150]. They will cooperate to evade host defenses, such as counteracting the prokaryotic CRISPR-Cas system (clustered regularly interspaced short palindromic repeats and associated proteins). They frequently cooperate and trade resources. For example, two different coliphages will synergistically partner. One might be strongly lytic but have a poor capacity to digest and penetrate the exopolysaccharide capsule of certain bacteria. The other can readily depolymerize the capsule but has a low lytic capacity to achieve co-infection on its own. That trading of resources need not be equal. Domingo-Calap et al. [150] report that phages can act ‘prudently’. Phages can act to reduce their short-term infectivity to improve long-term prospects through communication and cooperation with other phages. This contingent inter-communication supports their facultative lytic and lysogenic cycles.

Indeed, it may extend much further. Little is understood about multipartite viruses. It had been assumed that a simple viral unit and its associated capsid were the necessary requirements to complete the viral replication cycle. However, in multipartite viruses, the viral genome is not recapitulated within a single viral unit but is distributed within a co-existing viral population in partitioned separated segments. In this type of viral expression, separated segments infect different cells as ‘nanoviruses’ and rarely co-exist together within single cells. Somehow, they accumulate separably over time, act independently in different cells, and yet still manage to communicate sufficiently to recombine intermittently for full-scale reproduction. None of this fits our classical viral narrative, motivating a consideration that viruses, just like cells, can operate beyond the level of any individual cell and achieve their own form of multivirality that mirrors multicellularity [151].

The expression of these contingent behaviors governs the virus’s role within a target cell. It may be as an individual mutualist, parasite, commensal, or dependent on collective behaviors such as has been documented in poliovirus [152]. No matter its outcome, that relationship is essentially entwined and symbiotic, thereby shaping the evolution of the partners within a cellular ecology or holobiont [15]. Thus, such seemingly disparate outcomes from a viral infection can be seen as different expressions of virus-cell symbiosis, yielding varied outcomes, including latency, retroviral insertions permitting long-term viral replication, the favorable regulation of cellular genes, amplification of transposable elements, or superinfection exclusion [153].

Superinfection exclusion is the prevention of a secondary viral infection in the presence of a preexisting one. In the case of *Citrus tristeza* virus, a positive sense RNA closterovirus, one strain blocks another through the production of P33 protein of viral origin [154]. Although superinfection exclusion might be regarded as primarily aimed at protecting the initial virus by eliminating competition for target cell resources by another or reducing the chance of recombination or re-assortment on replication, characteristics of viral-cell symbiosis have also been identified. Therefore, superinfection exclusion can be viewed further as an aspect of dynamic equilibrium among viruses, virus-infected cells, and uninfected ones [155]. As a result, viral interference is not a simple matter of the production of a few molecules but an integrated dynamic of modulated viral virulence, lytic capacity, and cellular immune responses that all impact disease incidence and epidemiology [156].

This suite of complex inter-related, competitive and consensual biological outcomes is not merely the result of viral automatons and their defaults in continuous conflict with intelligent measuring cells. Instead, superinfection interference should be considered an expression of viral awareness as an instance of ‘swarm intelligence’ [157]. Thus, the decision matrix for denial of entry as co-infection must be properly viewed as a signaling event among individual agents in which the viral participants exhibit “intelligent swarm-based self-scattering behavior” [157]. Notably, this same type of swarm behavior is widespread across biology, including slime molds, insects, flocks of birds, schools of fish, and humans [158].

## 9. Viral agents as drivers of cellular biology and biological evolution

There is increasing recognition that viruses are not merely participants within ecosystems but have their own unique ecosystems that balance differing viral species, selected microbial strains, and cellular targets as potential partners [159]. We maintain that such ecosystems should be placed within a framework of contextual symbiosis among the four domains. Witzany [160] offers an encompassing perspective. An extensive panoply of viral elements (endogenous viruses, transposons, retrotransposons, long and non-long terminal repeats, long and short interspersed nuclear elements, group I and II introns, phages, plasmids) have co-evolved with cells and inhabit all genomes to such an extent that they should be considered ecological niches [160]. In those sites, they genetically engineer shifts, insertions, deletions, recombinations, and epigenetic controls to fully participate in evolution and cellular developmental processes in reciprocation with their own viral ecosystems.

Recent research with *Potato virus Y* illustrates the complex interactions between a viral infection cycle and plant cell machinery [161]. In addressing a viral infection, the plant activates a highly sophisticated and multilayered immune defense system. In the absence of overwhelming viral pathogenesis, the cellular response can be symbiotic balancing or the active deployment of the viral genomic complement as a tool with the potential for phenotypic outcomes. Thus, the range of intracellular viral behaviors represents a co-existing superimposition of possibilities that mirrors the general dependence of cells and viruses on information. Thus, neither overt viral pathogenesis nor superinfection exclusion is the most common outcome of viral encounters. In plants, mixed viral infections have been underestimated, and infective diversity is more the rule than the exception [162]. The diverse range of possible outcomes from viral infection and the commonality of viral symbiosis both represent contingent actions that require the reception, assessment, and deployment of information by viruses and their target cells. Such actions can be realistically placed within an engineering framework. Both viruses and cells participate in a contingent, information-based process through overlapping decision matrices, i.e., as a virocell.

Recent evidence supports that the viral capsid can provide functional benefits that extend beyond the originating viral replicon. A capsidless (+)ssRNA virus YkV1 has been shown to be dependent on an unrelated dsRNA virus, YnV1, for encapsidation and replication [163]. This too, represents instances of viral engineering capacities and even deemed a form of problem-solving.

If viruses are capable of engineering, then they should be able to use tools. Without question, viruses use cells as their tools, and reciprocally, cells use viruses as theirs, forming the explicit basis of viral symbiosis [14–16,164,165]. It has been conclusively demonstrated that viruses can use cells to their ends, being termed ‘puppet masters of bacterial hosts’ through their ability to alter bacterial metabolism toward their requisites [166]. In doing so, they can deploy capsid proteins to further their particular aims within that target cell context. For example, adeno-associated viruses can deploy capsid proteins and structural coating or uncoating to promote specific cellular interactions and recruitment of cellular tools through capsid trafficking, viral assembly, and genome packaging [167]. Through recruited cell-cell adhesion domains, viruses manipulate cells by inducing specialized cell-cell channels, permitting more effective intercellular viral transport and enhancing viral and cell-cell communication [7].

Within this later dynamic, vesicles are among the most useful viral tools. Virions disperse inside cells as single virions or in groups. One type of group dispersal is termed ‘collective infectious units’ that can achieve several forms: as a single virion with multiple genomes (polypoid virions), groups of free-standing virions that create a joint aggregate structure, or groups of virions within extracellular vesicles utilizing sections of cell membranes for their formation, or within lipid-membrane encapsulated inclusion bodies [168]. This latter structure is an example of viral tool-making that can take variable structural forms and is used by many different viral families. Lipids can also be viral tools, protecting against cellular antiviral immune responses in extracellular vesicle membranes, thereby enhancing replication and virulence [169].

In further support of the concept of viral engineering, it is argued that viral genomic modularity and the flexible deployments of capsid structural architecture and function should be considered agents of viral problem-solving and not products of stochastic processes. Ultimately, all such tools redirect cellular faculties to viral aims. Comparative analysis has demonstrated that the genomes of double-stranded DNA viruses display nodal modularity that aligns with distinct morphogenetic toolkits in which specific capsid proteins predominate. The evolutionary persistence of this genomic modularity across expansive viral families despite high rates of gene loss, gain, and exchange, supports extensive gene sharing throughout the double-stranded DNA virosphere [170].

The interaction of viral genomes with particular capsids permits viruses to engage with their target cells in multiple ways. The capsid in hepatitis B viruses is a double-shelled structure comprised of an outer envelope with three envelope proteins and an inner icosahedral capsid composed of a single core protein that surrounds the DNA viral genome (a small partially double-stranded relaxed circular DNA) [171]. DNA replication proceeds through reverse transcription of an RNA intermediate. In an intricately coordinated process within the cell nucleus, replication is carried out, yielding various products, including a DNA-containing entire virion and the release of two additional types of noninfectious sub-forms [171]. One of these latter sub-forms is an aggregation of viral envelope proteins that populate the blood of infected individuals at rates 100 to 100,000 times in excess over complete virions. The second type of sub-form is ‘empty virions’, i.e., genome-free virion capsid shells that consist of only the capsid and the envelope proteins but no viral genetic material. This sub-form, too, is at least 100 times more common than complete virions. How precursor DNA is excluded from these empty shells is unknown, as is the reason for their prodigious production. Yet, there is no doubt that the selective formation of these highly coordinated products is properly considered engineering.

Viruses need cells to replicate their genomes and must co-opt cellular biosynthetic capacities across a wide range of cellular compartments to accomplish this action, including the nucleus, cytoplasm, subcellular structures, or virus-induced rearranged membranes [172]. In turn, cells use viruses to supplement their immune defenses, transfer information, or trade resources among themselves. To that end, extracellular vesicles are now believed to represent a continuum of membranous particles, derivative of both cells and viruses, serving one or another or both, dependent on context [173,174]. The interdependent relationship is so close between RNA viruses and exosomes that enveloped viruses can be considered a form of extracellular vesicle. Further, it is unclear which was the origin of the other [175]. Indeed, they are formed and exported from cells via similar pathways. They share other aspects such as size, structural and biochemical composition, and the transport of bioactive molecules within cells. Furthermore, both extracellular vesicles and enveloped viruses can bind to the plasma membrane of target cells and, after direct fusion or after endocytosis, release their cargo into the cytoplasm to manipulate diverse cellular activities [60,174,176–181]. Extracellular vesicles emerge to be involved in numerous processes integrating multicellular organisms into coherent wholes, including cell-cell signaling and communication, regulatory messengers, sexual reproduction, synchronizing cellular clocks, sentience, cellular phenotypes, and assist in wound healing [182–192].

Further, extracellular vesicles are now recognized as regulatory messengers of cells, notably participating in the modulation of innate and adaptive immunity and cell fate [193]. Yet, when viruses and extracellular vesicles are co-participants as messaging components, which is regulated and which is the regulator? In either case, the result of the direct contact between viruses and cells is an extensive modification of both cellular and viral products.

Therefore, no matter our previous presupposition that host-pathogen dynamics predominate and viruses are obligatory parasites, there is sufficient evidence to sustain that viruses interact with cells within a co-respondent engineering construct. Target cell reprogramming is an explicit example of viral engineering. Reciprocally, cells can respond to viral incursions by deploying them, parts of them, or their engineered products for their own aims as Natural Cellular Engineering. Consequently, the relationship between cells and viruses is deeply centered within a continuous evolutionary narrative of cooperation, competition, and generally mutualizing co-existence based on viral-cellular co-engineering directed toward virocellular symbiosis.

Until recently, the extent of bacteriophage inhabitation of bacteria and archaea was markedly underestimated. The viral family, *Inoviridae*, is characterized by a unique morphology, genetic complement, and infectious expression [194]. Most reside within the cells as plasmids or endogenize within a target cell genome. Yet, virions are continuously released. Once believed to consist of only 56 members, recently developed machine-learning algorithms and metagenomic sequencing have revealed that *Inoviridae* is pervasive across bacteria and archaea, associated with nearly all bacterial phyla, and inhabits virtually all ecosystems [194]. These indwelling symbionts modulate cellular gene expression, participate in an inovirus-encoded toxin-antitoxin system, and express both synergistic (CRISPR evasion) and antagonistic (superinfection exclusion) interactions with other potential infectious-symbiotic viruses. Their ubiquity and persistence over evolutionary time spans argue for a new perspective on the relationship of the viral domain to cells. Obligate viral parasites should now be recognized as complex co-engineers with cells for mutual protection. Despite their highly destructive potential, viruses are primarily symbionts whose conjoining actions within target cells should be considered viral-cellular co-engineering. In biology, everything participates and reciprocates.

Indeed, that narrative of characteristic viral-cellular co-engineering has always been evident across evolutionary space-time. Contemporary stromatolites are examples of the residue of collaborative, complex microbial life as microbial mats. Fossilized ancient ones have been dated back billions of years, yet, they still match modern iterations. How microbial mats became calcified structures had been unknown. Recent research reveals that the process of lithification (carbonate precipitation) is based on codependent virocellular interactions that play a continuous role in global biogeochemical cycling [195]. In this process, symbiotic coalescent phages of cellular microbes stimulate carbonate precipitates, forming large spheroid aggregates constructed of nanoparticles that curiously resemble viral-like particles. Their collective deposition forms stromatolites as ancient evidence of a perpetual virocellular symbiosis [196].

## 10. Coordination between viruses and cells should be considered engineering activity

**Figure 3.**
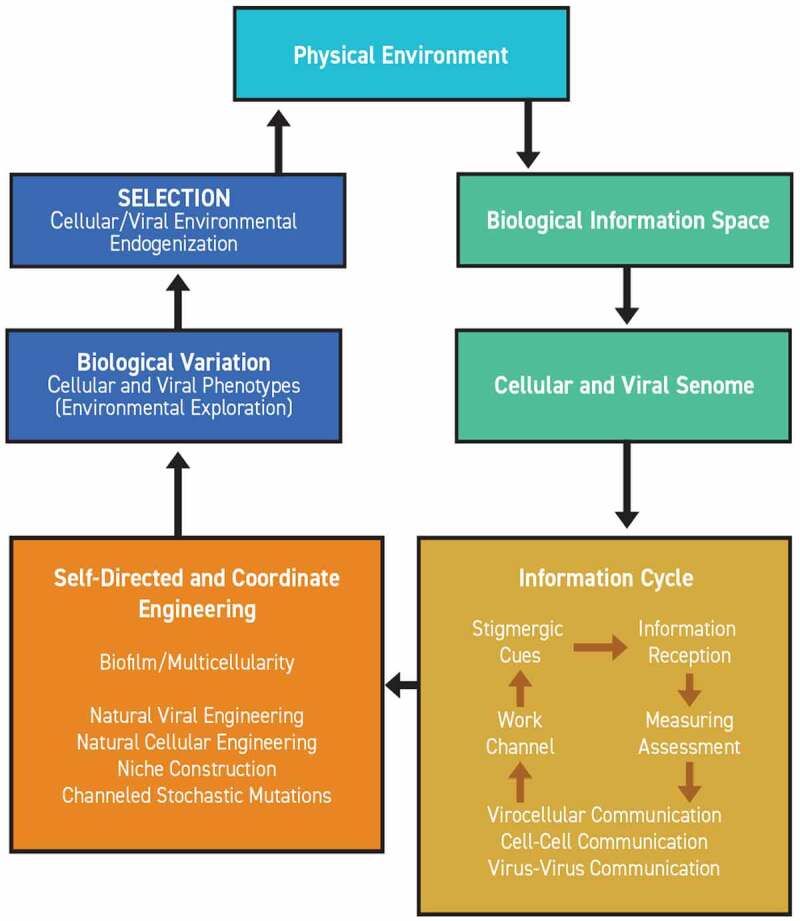
**Information management within Cognition-Based Evolution.**Biological information space is constituted by environmental cues which impact the senomes of self-referential cells and viruses. Information reception initiates a reiterative information cycle that permits viral and cellular natural co-engineering. Information is received, measured, communicated, and deployed, energizing an obligatory work channel reinforced by stigmergic cues. That information cycle is the backbone of Natural Cellular Engineering, Natural Viral Engineering, and niche constructions. Natural genomic editing and stochastic events are channeled aspects of the engineering cycle. These act coordinately to produce biological variations as phenotypic alterations for environmental exploration. Those variations are subject to filtering selection directed toward the continuous virocellular endogenization of the external environment.

The application of the term ‘engineering’ is not new in biological contexts. This frame has been previously applied to the cellular genome as both ‘natural genetic engineering’ and ‘natural genome editing’. The same conceptual framework has been applied to the developmental structure of microbial biofilms and multicellular eukaryotes as ‘Natural Cellular Engineering’ [34,35,37,39,40,129,130,160,197–204]. Once biological processes are placed into the context of engineering, these coordinated activities can now be properly perceived as an issue of information management [33,35] (Figure 3). A commonly used general definition of engineering is the “action of working artfully to bring something about.” Once cells are correctly understood as intelligent and measuring, all subsequent multicellular life must be regarded as forms of strategic information management. Information management depends on computational measurement and communication that permits collective effort with the potential for creativity. In these terms, cells are engaged in engineering as collaborative problem-solving in confrontation with environmental stresses, including unpredictable events. Accordingly, cells engage in Natural Cellular Engineering as information management and problem-solving at their scale. Viruses are active participants in these collective cellular efforts.

Cells are equipped with sufficient faculties to support engineering capabilities [205]. This capacity is doubtless present as cells are no longer regarded as automatons and are instead recognized as cognitive agents [19,20,22–24]. Indeed, cells display all aspects of conscious sentience [18]. Cells are intelligent, measure environmental cues, and communicate information assessments among themselves [34,37]. Through that measurement, they determine which tools to deploy to uphold their homeorhetic equipoise [39]. In so doing, cells demonstrate objective cognitive characteristics that seamlessly meld across scales [19,205]. This range of purposive action is sufficient to enable engineering within their scope, analogous to our own human interchanges. These actions include high levels of cooperation, collaboration, co-linkage, co-dependence, the trading of resources, and competition. At every scale, these behaviors require a linked ability to evaluate relevant environmental sensory cues to sustain effective search strategies to support targeted decision-making that might enable mutualizing niche constructions. In this manner, organisms reciprocate with environmental landscapes across successive ecological levels to yield evolutionary outcomes [206]. Notably, all these activities also require successful concordant engineering as mutualizing niche constructions to survive in hostile environments, just as is the case for human endeavors.

A claim that viruses engage in any form of natural engineering would entail providing evidence that viruses can substantially mimic cells in having some capacity for contingent decision-making. This perspective is supported by the documented social activities of viruses. Moreover, mounting information indicates that viruses can act in their own behalf by communicating and cooperating both outside and inside cells [135–137]. Consequently, these exhibited faculties should be considered as a form of limited contingent decision-making and problem-solving.

Engineering is the purposeful application of information and its directed communication to deploy available physical and energetic resources to produce results that meet specific functions. The suite of objective viral and cellular faculties and the landscape of interactions displayed through their collective should be regarded as symbiotic engineering. That coordinating ensemble includes measurement, purposeful communication, the flexible use of tools according to context, modularity, divisions of labor, and the adroit collective deployment of a large range of resources. Their coordinated yield is integrated functional outputs. Notably, those outputs depend on behaviors that specifically mirror engineering at our human scale, including cooperation, co-dependence, and competition. Cells are engaged in complex signaling processes guided by “syntactic (combinatorial), pragmatic (context-dependent), and semantic (content-specific) rules” [207]. Given the essential reiterative nature of evolutionary development, it is no longer surprising that our human engineering is self-similar and gratifyingly capable. Surely, our augmentative intellectual privileges have permitted our human stamp on our engineering processes, reflecting our creativity at our scale. Similarly, cellular cognition is objectively real but should not be judged only by our human faculties for abstract thought and reasoning [22]. The innate creativity of cells is real. We are their product [34,37]

The concept of tinkering in evolution is widely acknowledged and is pertinent to the concept of engineering at any scale [208,209]. Tinkering is an exploration of materials to make things, and as such, is one expressive form of engineering [210]. For example, in plants, root nodule symbiosis is a co-option of a portion of the mycorrhizal symbiotic machinery that enables conjoint signaling pathways between plant cells and their co-adapted microbiome. The development of these symbiosis-specific organs occurred 100 million years ago and required the layered co-option and deployment of orthologous genes to support and enhance symbiotic machinery that originated over 300 million years earlier. Cells can engineer and tinker productively because they can measure, communicate, predict, and deploy resources with purpose. Importantly though, as Jacob [209] correctly asserted, any comparison of natural selection to engineering is unsuitable. Natural selection has none of those cellular engineering properties and only acts as a post-facto filter of prior biological activity. Selection assures that those cellular measurements are correct within their obligatory context of continuously meeting environmental proscriptions [34].

Natural genetic engineering is the process by which cells accomplish extensive genome-wide organizations within a few generations and has been researched and refined for over twenty-five years [24,198,213]. Significantly, when engineering is identified as the prime mode of genetic variation, random genetic variations can no longer account for genetic re-configurations. Further, such variations need not be gradual [39]. A suite of genetic elements are accredited agents of variation in natural genetic engineering. These include transposable elements, plasmids, and varieties of RNAs that can participate in genetic editing in nonrandom patterns through integrated genetic regulatory systems [39,201,211,213].

A central concept of natural genetic engineering is that the genome is a read-write memory system rather than a fixed blueprint [214]. Cells continuously engineer flexible responses to environmental stimuli through modular and repetitive coding motifs, replication, transmission, protein expression, and genetic repair. As a result, genomes actively participate in self-modification through natural genetic editing and epigenetic imprinting that determine their ongoing biological expression. Significantly, viruses and viral agents are active players in genomic editing, acting as non-lytic symbiotic viral swarms that persist within cellular host genomes [212]. Most of these actions devolve through RNA agents such as transposons, retrotransposons, and small non-coding RNAs acting as consortia which can be co-opted by cells as adaptations in key cellular processes.

**Figure 4.**
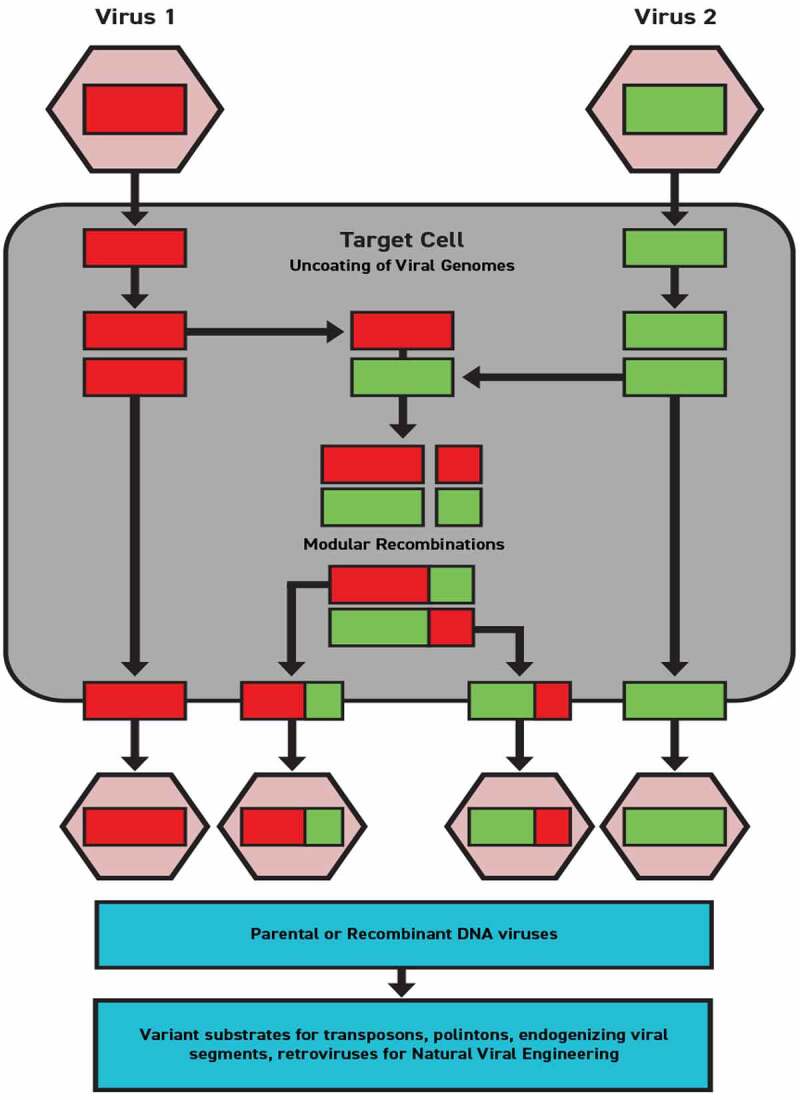
**DNA viral exchanges for Natural Viral Engineering**.Viruses can exchange modular genetic segments, which can participate in natural genetic editing as ‘plug-in cassettes’. These modular genetic segments can represent substrates for various retroelements that participate in natural viral-cellular engineering, enabling exon shuffling or altering *cis*-regulatory sites. These exchanges contribute to the pool of mobile DNA as part of the fluid trans-kingdom transfer of genetic material.

The modularity of mobile genetic elements as ‘plug-in cassettes’ that can modify genetic code is critical to genomic editing [214,215]. Modularity enables exon shuffling or altering *cis*-regulatory sites, accommodating HGT or genetic duplications. As one of the consistent mediators of natural genetic engineering, mobile DNA is a crucial aspect of the fluid trans-kingdom transfer of genetic material [216] (Figure 4).

Natural Cellular Engineering encompasses natural genetic engineering since genes are tools of cells and viruses [34,37,40]. Additionally, Natural Cellular Engineering identifies cells as intelligent agents capable of measuring ambiguous environmental cues to flexibly uphold themselves. Genomic rearrangements and their read-write mechanisms are crucial aspects of the continuous maintenance of cellular homeorhesis in confrontation with environmental stresses as an essential facet of ecology-wide multicellularity. Thus, both microbial biofilms and multicellular eukaryotes as holobionts are the result of Natural Cellular Engineering, NVE, and natural genomic editing, which coordinately produce linked cellular ecologies and phenotypes directed toward the maximization of effective environmental information. These products permit the survival of all participating cellular constituents through fluid cell-cell communication [20,34,35,129,199] .

The concepts of natural engineering and niche construction apply across living scales. Similarly, the role of symbiosis is central to biology. Indeed, endosymbiosis is acknowledged as the hallmark process enabling the eukaryotic cell [217]. It has been previously argued that the true nature of that symbiosis should be understood as consistently directed toward the perpetuation of each of the four domains: Prokaryota, Archaea, Eukaryota, and the virome [34,199]. Within this framework, the products of Natural Cellular Engineering can now be recognized. Biofilms and holobionts are engineering products in response to environmental forces for perpetuating the four domains across evolutionary space-time. There is evidence of the firmest kind for this proposition. Only these four biological entities have continuously existed for billions of years. The virome is a vital intercessory among the three cellular domains. As such, viral actions extend beyond pathogenesis, encompassing a fuller dynamic that should be understood as a form of communication and mutual information management among all cellular domains with viruses as intermediaries [199]. Alive or not, within cellular boundaries, viruses are capable of limited sorts of engineering to achieve their own ends. Thus, viruses are reciprocating participants with cells, acting in a nonrandom manner toward generally symbiotic and mutualizing goals.

Forterre [55] formulated the ‘virocell’ concept, emphasizing the intracellular viral metabolic and disassembly-reassembly phases that utilize cellular resources for viral reproduction. The virocell concept views cells and viruses as an entwined co-relationship that transforms an infected cell into a ‘virocell’ as a novel cellular organism. In that frame, viruses originate from ribosome-encoding cells in which both viruses and cells participate in intracellular protein production. Bi-directional viral-cellular HGT becomes a function of a full range of virocell activities. In this context, viruses are critical proactive participants in a symbiotic intermediate state, which has been termed a ‘carrier’ state or persisting ‘riboviro-cell’ [41]. Thus, the virocell metabolism is a unique metabolome jointly determined by the cell’s metabolic and immune capacities and the introduction of a suite of auxiliary virus-encoded metabolic genes [218]. It does not matter that there is a relative paucity of virus-encoded auxiliary metabolic genes. In concert with their capsid and envelope proteins, their weight is sufficient to impact individual cells substantially, and further, their intimate mutualizing coordination warrants being placed within a co-engineering framework.

**Figure 5.**
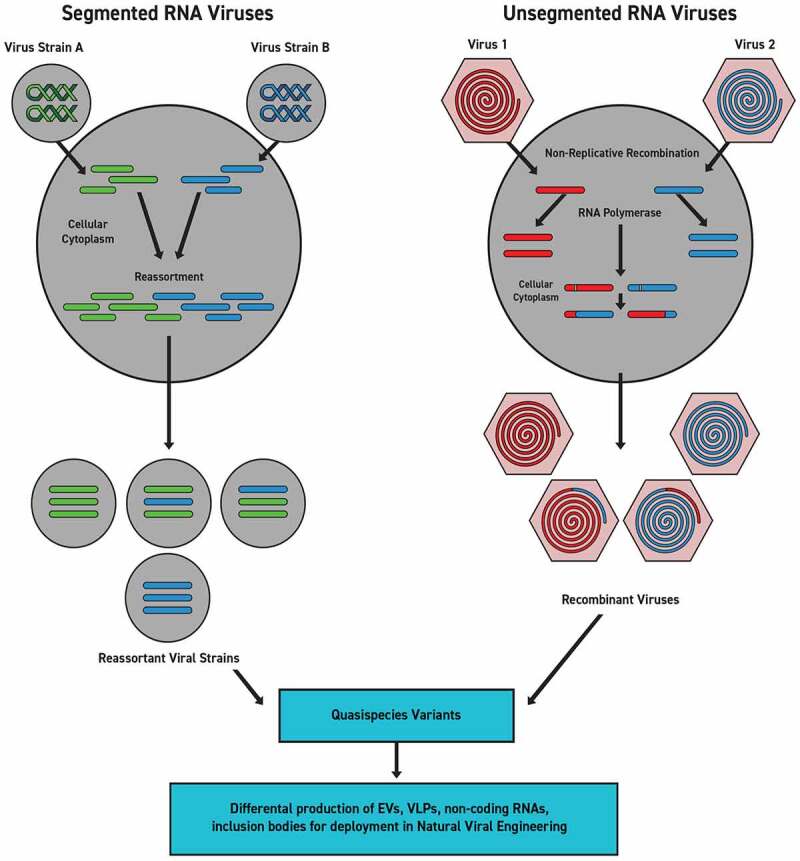
**RNA viral reassortment-recombination in Natural Viral Engineering.**Segmented and unsegmented RNA viruses undergo reassortment or recombination within cells, producing reassortment-recombinant viral strains as new variants. These vary further as quasi-species, providing a flexible means of evaluating the cellular environment. All can participate in the differential production of substrates for NVE as VLPs, non-coding RNAs, inclusion bodies, or as participants within EVs.

Beyond their intra-cellular actions, viruses engage in engineering activities outside cellular boundaries. For example, it has been revealed that viruses can form collective infectious units for the joint delivery of multiple genome copies to target cells for co-infection by traveling as a group [219]. This collective transmission proceeds by several means involving virion aggregation and specific extracellular components, encasement in protein matrices, inhabiting lipid vesicles, within occlusion bodies, or aggregating on cell surfaces. Certain issues are clear despite many unknowns about any putative selective advantages or disadvantages. Viral group dispersal is widespread across the virosphere, involving many different viruses (enveloped and non-enveloped, either segmented and non-segmented, [(+) ssRNA, (-) ssRNA, dsRNA, dsDNA]), may be of similar viral types, or formed from multiple viral types with differing genomes (Figure 5). This collective action is a form of cooperative activity that requires interviral co-engineering. As it is extracellular, it can be correctly deemed Natural Viral Engineering and not just an aspect of natural cellular activity.

The long-standing conception of viral dynamics acknowledged that viruses co-opt cellular machinery but presumed this action was predetermined autonomic templating. Fresh analysis argues that this perspective is unduly limited. Others have described the essentials of this relationship in terms of a critical planetary-wide viral symbiosis [10,11,16,17,164,165]. As such, this type of symbiosis is well-suited to a cohering framework of co-engineering. Those distinctive viral engineering products, both within and outside the cell, represent the crux of the terms of that relationship. What is not in doubt is that virocell metabolism and its encoded metabolic products help to shape aquatic and planetary environments, either directly or through giant viral intermediaries [95].

Although viruses have many recently discovered contingent capacities, they still have many limitations. How might viruses with proscribed faculties, alive or not, engage in the complexities of engineering? In answer, the issue of stigmergy is illuminating. Stigmergy is an active means by which cells and macroorganisms coordinate according to simple dynamics [20,35,129,220]. In stigmergy, one action cues the next. The output of a first action becomes a conditional aspect of the process workflow that follows. Within a stigmergic flow, it is sufficient that the individual agents share a general direction. Stigmergy has been proposed as a means by which a set of organisms can spontaneously coordinate. Significantly though, if flows are interrupted, there must be some kind of reciprocal negative feedback, termed ‘error-controlled regulation’ that terminates that differing direction. The advantage of stigmergic systems is that they do not require prediction, anticipation, memory, or knowledge of a specific aim on the part of any of the individual participants. It is enough that there is a general shared preferential direction and an ability to offer a contradicting influence in certain circumstances. The results are forms of parallelism and sequential actions critical to engineering processes. Although it is debatable whether viruses have any capacity for anticipation, it is quite evident that they do follow cues and coordinate sufficiently to enable group dispersal and collective action for co-infection. Nor is there reason to doubt that viruses have some states of preference. The predilection of certain viruses for specific tissues and organs that underlies one essential aspect of medical diagnosis represents direct evidence. Certain viruses prefer specific hosts, cell types, tissue ecologies, or organs and can achieve differing states within the same organisms in different cells.

Naturally, the validity of any concept of viral engineering would be heightened if viruses have memory to contribute to their mix of coordinating faculties. Do viruses have memory? Cells have viral memory based on vertically transmitted innate and trained cellular immune responses [221]. Could that memory system reciprocate with viruses? Although that notion would have been swiftly dismissed a few years ago, recent research has revealed that a major widely distributed protein involved in cognition, Arc, strongly resembles and acts like a viral protein [222]. The Arc structure is similar to the HIV retrovirus. Arc’s protein sequences have coding regions similar to viral capsids. Such capsids form a critical tool for viral infection but also mediate cell-cell communication through RNA transport. Data suggest that the evolutionary origin of Arc is from ‘gypsy’ retrotransposons which are integrated in genomes across the animal kingdom but can independently replicate. Arc is a capsid-forming viral-like gene that has apparently been exapted from viral origins, serving a crucial role in intercellular communication, cognition, and memory. It might be expected that these same genes might have a use within the originating virome that, in some manner, resembles its action among cells.

Any concept of viral memory need not carry the connotations that we might ascribe to our type of cognition. Viral memory may be unlike that of cells, plants, or animals. It need be sufficient merely to permit modifiable contingent actions in support of efficient parallelism and sequential actions to effect those basic engineering capacities that have served adequately over evolutionary spans. There is evidence that this type of proscribed idiosyncratic viral memory might exist. Based on experimental evidence and modeling of human immunodeficiency virus type 1, viral quasispecies may possess a modifiable molecular memory of their evolutionary course [223]. This form of memory works to their advantage. Viruses and bacteria appear to carry memories of prior patterns of infection [224]. It is believed that viral memory accounts for differential patterns of infectious transmission between the sexes that has never been previously explained (females infected by males or males infected by females have higher mortality from measles, chickenpox and polio than same sex transmission).

Data inform us that repetitive molecular sequences reemerge in quasispecies that had remained cryptic over long periods, believed to represent quasispecies recovery of a preexisting genomic capacity as a form of memory. This is not the only instance of potential viral memory. For example, in the case of the foot-and-mouth disease virus, RED is a deleterious gene that should have been expunged but remains present [225]. It has been proposed that it persists as a form of population evolutionary history as memory. In this frame, viral quasispecies can be understood as variant subsets of viral genomes that cycle between minority and majority status over the course of planetary evolution [226]. In theory, some quasispecies are the renewed appearance of memory genomes based on a memory marker (an internal oligoadenylate tract of variable length) that are potential sources of high fitness, though typically remaining cryptic. In effect, quasispecies are forms of memory within the virome as they participate with the three cellular domains across endless planetary cycles.

Reemergence of prior viral iterations with higher mutant frequencies can be thereby ascribed to quasispecies memory and are not simply the result of random occurrences. Prior configurations that contribute to adaptation have deterministic features [224]. Indeed, this has been observed experimentally [152]. The concept of sequence space has been applied to viral genomes to explain the high mutation rates of RNA viruses throughout evolution. Sequence space can be regarded as representative of information space, strengthening the case for the occurrence of viral quasispecies variants as a function of nonrandom viral memory, at least in part [152]. Given the immense number of potential variants that could arise even from a small genome, the viral sequence space concept proposes that mutant variants occupy a mutant ‘cloud’ or ‘swarm’, constrained through a theoretical mutational fitness landscape. Depending on environmental changes, there are multiple places in the ‘cloud’ from which ‘adaptive walks’ might initiate [152]. NVE proposes further. Quasispecies are the viral means of exploring information space through a variety of viral phenotypes. The perspective that viral quasispecies should be placed into the context of phenotype has been previously introduced [225]. Further, Villarreal and Witzany [227] argue that the entire concept of quasispecies requires reconsideration. Instead of an individual variant, quasispecies properly represent collective consortia of RNA stem-loops as cooperative modules, acting as members of a co-linked social group in stem-loop societies. Viruses, viroids, mobile genetic elements, and ribozymes are RNA stem-loop secondary structures that exert stem-loop phenotype as biological variation.

Three concepts deserve further examination in support of the contention of viral engineering: viral factories, virocells, and sociovirology. The term ‘viral factories’ describes how viral clusters in perinuclear or cytoplasmic foci disassemble and assemble by recruiting certain specific cellular organelles, such as mitochondria and cytoskeletal structures. Various cellular proteins are produced through coordinated viral action to enact the viral replication cycle [228,229]. Positive-strand RNA viruses rearrange intracellular membranes into ‘organelle-like RNA replication factories’ [230]. This process involves numerous virus-induced vesicles, partially derived from endoplasmic reticulum membranes close to or at the site of viral assembly [230]. As part of this process, inclusion bodies, termed ‘viroplasms’, represent highly organized viral factory sites. These viroplasms are directed toward viral genome replication and capsid packaging, providing assembly intermediates to the endoplasmic reticulum [231]. These inclusions also serve as protection against cellular antiviral defenses [232]. Cellular microtubules in both plants and animals are crucial to the process, contributing guidance and anchorages of these viral factories as they traffic across the endoplasmic reticulum/actin network [233]. This complex process represents engineering since the operations define the pertinent viral form. Claverie [234] proposed that the process itself matters most. Indeed, the “virus factory should be considered the actual virus organism when referring to a virus” [234]p. 110.4).

The virocell concept concentrates on the intracellular phase of the viral reproductive life cycle, regarding it as a new type of cellular organism [235]. The virus and the cell begin as two separate entities. Yet, once the virus gains intracellular entry, a new co-respondent entity, the virocell, develops, which combines a capsid-encoded entity with a ribosomal one as a riboviral-cell [235]. This can subsume several distinct forms and endpoints ranging from long-standing symbiosis to death of the virocell. Yet, there is a uniting confluence among these differing outcomes. All are products of co-engineering, consensual or not, between the virus and its target cell. Thus, evolutionary development shifts from a random process to one that matches our own engineering processes as products of collaborative intelligence from which creativity can spring. Context and meaning within biological organizations drive cognitive behaviors in living organisms that reciprocally feed back from all cellular and viral participants to impact the genome, permitting flexible adaptation [236].

Assuredly, natural environments offer myriad random inputs. Experimental evidence indicates that both their amplitude and predictability have a large impact on their selective influences [237]. Others have maintained that stochasticity steers evolution by directly shaping selection pressures [238]. Our contention is that stochasticity becomes a distinct positive within an engineering framework. Every engineering enterprise encounters unexpected random events. Pertinently, these generally represent endemic ‘noise’ in any biological system. However, intelligent agents make use of these random events through the ‘harnessing of stochasticity’ [239]. Cells use their sensory apparatus to discriminate those high amplitude events that are outliers to the general pattern of random noise that might represent an input signaling an alteration of a previous environmental trend. Necessarily, this represents a prediction that is ‘harnessed’ by cells as a useful environmental cue that should be measured and potentially deployed within the viral-cellular engineering cycle [34,37]. In this manner, erstwhile random inputs become a useful feature of cellular engineering, mirroring how serendipity can be occasionally leveraged to favor human engineering.

## 11. Are co-partnering viruses alive?

At first consideration, viral participation in engineering might strenuously argue for their living status. After all, engineering is a measuring process requiring some capacity for assessment that can be directed toward nonrandom problem-solving. Might a virus be able to attain living status inside the cellular milieu to become a co-engineering participant in consensual and non-consensual outcomes? No one knows, but alive or not, viruses display sufficient faculties to deem them capable of participating in engineering processes, at least within cells. Although whether viruses are alive or not is undoubtedly important, it weighs more that we understand their engineering capacities and its precise limits, as the virome has had a dominating role in the evolution of life.

The virocell concept makes it problematic to regard viruses as merely non-discretionary particles rather than participant organisms [240]. The emerging science of ‘sociovirology’ poses an equivalent challenge to any assumption that their actions can be modeled only as automata. It is known that individual virions fail to establish any co-engineering foothold in target cells [241]. Successful cellular co-habitation depends on collective intracellular viral units. Intracellular viral persistence is mediated by many newly identified viral infective units, including polypoid virions, aggregates of virions, virion-containing proteinaceous structures, and virions within protected vesicles [241]. Viruses cooperate and compete in collective units just as cells do [242]. Indeed, such virus-virus interactions are pervasive [136]. If two viruses gain entry into a target cell, they may either cooperate or compete, and there need be no necessary correlation with any identifiable genetic complementarity [136].

Moreover, viral sociality also conforms to an identifiable set of biological principles that dominate the living frame, constituting aspects of both biological and social engineering. Agnati et al. [243] argued that biological complexities rest on three conceptual tools: mosaic reiterations, self-similarity, and a principle of Biological Attraction. The mosaic principle reflects that the same elements can be reiteratively deployed to create different complex patterns. Self-similarity argues that the same rules for carrying out logical operations remain constant at all scales. The Principle of Biological Attraction directs that each living agent creates a ‘sphere of influence’ around itself that modifies the environment, exerting an inherent drive for spontaneous attraction. This drive promotes the merging of like-kind entities at scale that can generate successively higher levels of organization. These principles operate concurrently at the molecular, cellular, and supracellular levels. From within those principles, genes form genomes, cells combine into linked tissue ecologies, and multicellular organisms enact complex social structures that enable cooperation and discipline competition. There is good evidence that the virome should be included in this same encompassing narrative insofar as viruses demonstrate a robust social structure [168].

Recent research supports this conclusion. Viral escape from interferon-based innate immunity has been demonstrated to be a social process [147]. Domingo-Calap et al. [147] conclude that fundamental viral social rules exist, including cooperation, communication, conflict, and cheating. All of these impact the sharing of resources and the evasion of antiviral defenses. Indeed, viruses have been placed into a conceptual framework of social evolution theory to better explain the evolution of viral conflict and cooperation [136]. These dynamics can be highly complex. The trading of phage products such as capsids or structural proteins can be characterized as operating within social dynamics with sufficient nuance to be akin to such familiar social categories as the Prisoner’s Dilemma [150]. Even genetic exchanges appear to involve aspects of sociality. Multiple genetically distinct poliovirus strains can congregate to swap genes or gene products to enhance infectivity [149]. Further, rotavirus and norovirus clusters can travel together between cells as clusters in extracellular vesicles, affording protection and increased infectivity for both [244].

A surprising level of interviral communication exists among intracellular viruses, referred to as ‘chit-chat’ mediated by a six amino acid long protein, arbitrium, the Latin word for ‘decision’ [149]. It is now clear that viral communication is highly complex and fluid, functioning in an analogous manner to bacterial quorum-sensing. Through these means, viruses cooperate to co-infect cells, spread, determine lysogeny vs. lysis, and collapse or evade cellular antiviral immune defenses [245]. Wide-array peptide-based communication systems, similar to arbitrium, are used by phages to explore lysogeny decisions which are then executed by an antisense RNA as a regulator of the lysogenic state [246].

Viral communication ability can encompass an ‘understanding’ of a cell’s communication processes. A quorum-sensing receptor of a phage can detect host-produced autoinducer quorum sensing signal molecules that cues the phage lytic-lysogeny fate switch [247]. Moreover, the same signaling pathways are used by phages to disseminate among bacteria, a process that functions in the dispersal of *Vibrio cholerae* in human infections [247]. Based on the autoinducer 3,5-dimethylpyrazin-2-ol (DPO), that same quorum-sensing system is used for interactive communication across the eukaryotic, bacterial, and viral domains. This type of cross-talk is also deployed for other purposes. For example, among genetically diverse mammalian enteric RNA viruses, bacteria-mediated viral co-infection facilitates viral genetic recombination as a viral repair mechanism [248]. These bacteria-virus interactions are thought to be a cooperative means of increasing infectivity at the site of potential infection.

The extent of viral cooperation is just beginning to be explored in depth. However, the level of collaboration is sufficiently complex that it can be regarded as a form of altruism [148]. Bacteria can deploy a CRISPR-Cas adaptive immune system to protect themselves from phages [249]. Some phages will infect a cell and produce an Acr protein complex to weaken CRISPR-Cas but will be destroyed in the process. This leaves the cell immuno-depleted, which permits the successful infection of the cell by other phages in the population.

The exact boundaries for defining whether something is living or not remain highly debated and beyond our current tools. Some have advocated for defining life as a process rather than a material set. Torday and Rehan [250] and Witzany [251] regard life as communication. De Loof [252] agrees and further asserts that the point of any living communication is problem-solving. In that later regard, since viruses can cooperate to engineer, it defaults that this must also represent a form of problem-solving at scale. Dupré and Guttinger [253] argue that viruses should not be considered living ‘things’ in a conventional sense but ‘living processes’ which are part of an integrated. Interconnected, and collaborative living system. Forterre [52] asserts similarly, regarding viruses as biological entities which are living precisely because they are inseparably involved in the living process. If correct, then, there is no doubt that viruses fulfill it. Viruses and capsid-less viral elements have been crucial to all the major evolutionary transitions, facilitating the trading of ‘public goods’, and driving multicellular complexity [254]. Perhaps, there are no exact borders between living and non-living entities. Instead, there may simply be an evolutionary continuum that is arguably exemplified by giant viruses [255]. It might need to be acceded that viruses occupy a contingent zone that does not meet our desire for absolute categorization. Consequently, we contend that it is best to recognize viruses as entities ‘sufficiently alive to engineer’ as a liminal form of life whose range of action is context-dependent.

## 12. The concept of viral-cellular engineering impacts evolutionary development

In several respects, the NVE concept forces a reconceptualization of evolution beyond conventional Neo Darwinism. It has been previously argued that the broadly accepted dogma that evolution is dependent on random mutations for generating phenotypes is untenable. Accumulating research has uncovered that surprisingly robust mechanisms exist to control DNA mutational errors and chromosomal degradation. Stochastic DNA damage, such as ‘programmed’ DNA breaks during metabolic processes, is rapidly repaired to safeguard genomes [256]. Furthermore, selection, as conventionally appraised as a driving force, is not a dominating factor [257]. Wright [258] emphasizes that environmental influences shape evolutionary outcomes in nonrandom patterns through stress-induced transcription. Further, other nonrandom means of generating biological variation have been described in detail [39,259].

Crucially, engineering is neither random nor haphazard. Careful analysis of tripartite RNA genomes by polymerase chain reaction indicates that RNA segment re-assortment is not random [260]. The integration of HPV into cervical cancer cells is governed by a nonrandom distribution of integration loci [261]. There are nonrandom, relative ‘hot-spots’ for recombination in HIV that encourage epidemic spread [262]. Phylogenetic analysis shows that Influenza A genetic re-assortment is not random as only isolated genetic segments are involved [263]. Further, our human efforts at bioengineering indicate that retroviral vectors can be deployed for gene therapy. Such engineering is predicated on evidence that the integration of retroviral vectors follows a nonrandom pattern in mammalian cells [264].

Instead of basing variation on Neo-Darwinian random replication errors, biological variations can now be better understood as arising from natural cellular and viral engineering and their co-engineering products as the actual source of variations. Genetic variations, such as those that yield adaptive immunity, were not the result of random DNA replication but are contingent responses to viral incursions shifting a pliant read-write genome [265]. This highly coordinated process yields engineered products which are subjected to selective pressures. Moreover, that output is based on information and communication to permit engineering as problem-solving.

An engineering framework also transforms our understanding of phenotypic expression, decidedly shifting it from the accumulation of random genetic errors to representing nonrandom biological problem-solving to meet environmental stresses. It has been previously defended that phenotype is a cellular means of exploring the contemporary environment to enable the acquisition of epigenetic experiences that return for adjudication in the unicellular zygotic phase and embryogenesis [129,199,266]. In that same context, it has also been argued that phenotype is the means by which multicellular organisms explore information space [34,35,37,40]. It is now asserted that the deployment of viral quasispecies is an embedded feature of the viral engineering toolkit representing its own form of phenotype, either as individual variants or cooperative consortia. In this frame, viral actions can be considered analogous to how Natural Cellular Engineering produces tissue ecologies as cellular phenotype. Thus, quasispecies represent a means to explore sequence space as information space as their adaptive walk [152]. The resulting quasispecies consortia represent a viral phenotype that evaluates the environment and receives viral epigenetic impacts based on its cellular experiences. Just as eukaryotic gene expression is partially regulated by epigenetic influences, some of which can suppress viral gene expression, viruses adapt through their own epigenetic measures. For example, viruses can suppress their own epigenetic silencing during lytic replication or in their attempt to establish latent infections through a series of mechanisms. These are termed ‘epitranscriptomic modifications’, regulating viral gene expression [267]. These viral epigenetic modifications modulate the replication process, affecting virions, capsid and envelope production, and the tools of a cellular target. Thus, NVE and Natural Cellular Engineering are reciprocating aspects of a co-engineering partnership that forms the essential core of viral-cellular symbiosis, yielding biological expression either as microbial mixed-species biofilms or the cellular tissue ecologies that comprise holobionts.

Given the remarkable number of mutualizing cues and facilitators required between viruses and cells that enable both assembly-disassembly, a supporting, coordinating architecture developed in a nonrandom manner is required. This assertion can be further advanced based on the complex dynamics of instances of synergism in viral co-infection of the same cell [268]. In addition, multipartite viruses have genomes that are segmented and encapsulated among several different capsids that can be independently transmitted [108]. Yet, each must coordinate within separated target cells to complete their life cycle through remarkable intricacies.

The virome lies at the heart of information transfer as a major participant in horizontal and vertical heritable transfers, primarily through the infectious spread of mobile genetic elements [269]. Previously, this process has been framed as competition in Red Queen co-evolutionary dynamics [274]. NVE proposes that this process should be re-envisioned as consistent co-engineering on a planetary-wide scale directed toward continuous mutual solutions to environmental stresses. Those solutions are ever and always within the terms of the perpetuation of each of the Four Domains across evolutionary space-time. Both viral quasispecies and macroorganic phenotype are enjoined in a reciprocating exploration of the environment by returning contemporary cues to these endlessly enduring forms. It is argued that their perpetuation is a substantial affirmation of that assertion.

Some have asserted that since viral lineages lack structural continuity and membrane heredity, they should not be considered living forms [65]. Yet, paleovirologists have examined the evolution of hepadnaviral fossils in bird genomes and identified substantial differences between the prior estimates of viral sequence mutation rates and those that can be identified on an evolutionary basis [270]. For example, among hepadnaviral fossils in bird genomes, sequences remain up to 75% identical to contemporary hepadnaviruses despite an evolutionary time frame of 19 million years. This shift represents a thousand-fold diminution of mutational variation than would be expected based on previous estimates. This apparent paradox can be resolved by accepting that viral quasispecies are explorations of a limited potential sequence space cluster that directs toward biologically functional viral phenotypes. In effect, viral species tend to type, and reiterate those types and revisit them over long-term environmental cycles. Quasispecies are tools of long-term viral survival and they are repeatedly revisited as productive implements within the viral tool chest.

Viral-cellular co-engineering is a direct part of an overarching planetary ecology exerted at multiple overlapping levels and continuously enacted as cellular-viral symbiogenesis [10,11,164,199]. That process of symbiogenesis is epitomized by viral persistence as the asymptomatic colonization of species-specific targets as an essential aspect of viral evolution. Studies of marine environments have concluded that the virome serves as a reservoir of novel genes that move globally, participating in extensive gene swapping to manipulate marine environments by permitting successful cellular adaptations [2]. It has also been asserted that cell-cell communication is life’s critical buttress. Indisputably though, cell-cell communication is information transfer [20,35,37,148]. By re-conceptualizing the role of the virome in the context of their co-partnership with intelligent cells, the primacy of the role of information transfers crystallizes. The intricately coordinated holobionic form of life is ‘engineering’ since viral exchanges at all levels, virus-to-virus, virus-to-cell, or cell-to-virus, are information transfers embedded within the information management systems of the cellular domains. The virome is its essential accessory [20,40,68].

## 13. Conclusion

The 21^st^ century has benefited from striking advances in virology. Yet, their evolutionary implications have been reflexively placed within the familiar constraints of Neo Darwinism and its dual assumptions that viral mutations are random and competitive selection is paramount. Within that narrative, viral interactions at all scales are generally centered in terms of host-parasite dynamics. Cognition-Based Evolution proposes differently. Alive or not, viruses are reciprocating cellular partners. Together, they are co-engineering participants of the cellular ecologies that comprise biofilms and holobionts. Consequently, viral interactions extend well beyond pathogenesis and are better framed through nonrandom symbiotic terms. Cells co-exist through cooperation, collaboration, co-dependence, and mutualistic competition, which sustain multicellularity as biofilms and holobionts. These are the same interactions that frame viral-cellular symbioses. At all times, the full balance of these continuing imperatives among cells and viruses meets a single objective, observable, and consistent planetary narrative – the perpetual co-existent survival of each of the Four Domains. In this narrative, viral parasitism is real and can be problematic for cells and macroorganisms. Yet, in planet-wide ecological and evolutionary terms, viral persistence as latency, symbiosis, and endogenization are much more consequential than lysis [271].

In Natural Cellular Engineering, the multiplicities of phenotypes which characterize macroorganic life are the cellular means of environmental exploration to assure perpetual cellular survival [35]. For viruses, quasispecies, recombinations, and modular exchanges to create new viral forms serve a similar purpose. These variants are directed toward the continuous exploration of information space to assure their survival. In CBE, biology is based on information management. Thus, Natural Cellular Engineering and Natural Viral Engineering can now be further understood as conjoining means of exploring information space. Pertinently, when these are placed in their framework in which engineering is the selective deployment of information, biological and evolutionary development can no longer be considered random.

CBE stipulates that viruses and cells represent tool kits for one other, each with different rates of variation to meet individual environmental stresses. Just as Prochlorococcus and their cyanophages demonstrate a reciprocating symbiotic relationship, slowly evolving cells and rapidly evolving phages are reciprocating reservoirs of genetic and proteomic potentials to meet environmental stresses at all ecological levels, including holobionts. All are instances of viral-cellular co-engineering to assure their mutual perpetuation. Both cellular and viral phenotypes are a bioactive expression of exploring the environment as information space in the perpetual task of continuous successful environmental assimilation. Notably, this endpoint is exactly equivalent to selective advantage as an active filter of preceding potential viral-cellular solutions to environmental stresses. Toward that end, it has been previously proposed that evolution might uncover and exploit ‘underlying regularities’ upon which novel solutions might be grafted in a process likened to ‘learning principles’ [272]. We concur, directly arguing that this advantaged result is based on the the consistent interactions of viral players and cognitive cells, each with capable memories at scale, and reciprocating in continuous confrontation with environmental flux over evolutionary space-time.

Consequently, a fuller understanding of the role of the virome requires a paradigmatic shift. In particular, a reconceptualization of viruses from foes to generalized friends is required. Viruses and sub-viral particles are critical components of cellular phenotypic plasticity and successful adaptation through intimate symbiotic partnerships across the cellular domains [13,273]. Viruses are crucial linchpins. They are essential for maintaining planetary biodiversity and its ecosystems as full co-participants within evolutionary networks based on sharedconserved genes and fluid and reciprocating cross-kingdom exchanges [68,275].

Whether deemed alive or not, the virome demonstrates the ability to engage in limited coordination, communication, and collaboration. This contingent dimensionality is sufficient to be considered problem-solving, akin in type to that within the cellular domains. By implication, then, viruses can measure their context within their proscribed means along with cells. Since viruses can measure and communicate, they can engage in limited engineering as a discriminatory assessment of information and its management. The object of that engineering is straightforward. Viral-cellular symbiosis is co-engineering directed toward continuous environmental complementarity for all participants. Their directed products are biofilms and holobionic mutualized niche constructions, such as ourselves.

What, then is a correct contemporary definition of viruses? Viruses are a liminal form of life capable of co-engineering in the context of cells. That mutualizing engineering should be regarded as cross-domain problem-solving through the use of communicated measurements. These reciprocations are contingent co-engineering, representing a previously unheralded glue that assures either planetary survival or extinction among all living forms.

In CBE, evolutionary development is placed within a cohesive framework of natural viral-cellular engineering and mutualistic niche constructions as an ongoing perpetual reciprocating symbiosis among all the cellular domains and their viromes.

## Author Contibution

William B. Miller, Jr: Writing. figures -review and editing. Arthur Reber: Review and editing. Perry Marshall: Review and editing, figures. Frantisek Baluška: Writing- review and editing.

## Abbreviations

CBE: Cognition-Based Evolution; NVE: Natural Viral Engineering; VLPs: virus-like particles; HGT: horizontal gene transfer; MITEs: miniature inverted-repeat transposable elements; NCLDVs: nucleocytoplasmic large DNA viruses; sRNA: small RNAs; HadV-D: human adenovirus D; UCSs; universally conserved segments; ICEs: integrative and conjugative elements,
